# The chromosome-level reference genome of *Coptischinensis* provides insights into genomic evolution and berberine biosynthesis

**DOI:** 10.1038/s41438-021-00559-2

**Published:** 2021-06-01

**Authors:** Da-xia Chen, Yuan Pan, Yu Wang, Yan-Ze Cui, Ying-Jun Zhang, Rang-yu Mo, Xiao-li Wu, Jun Tan, Jian Zhang, Lian-an Guo, Xiao Zhao, Wenkai Jiang, Tian-lin Sun, Xiao-Di Hu, Long-yun Li

**Affiliations:** 1 Chongqing Academy of Chinese Materia Medica, 400065 Chongqing, China; 2 Chongqing Engineering Research Center for Fine Variety Breeding Techniques of Chinese Materia Medica, 400065 Chongqing, China; 3 Chongqing Sub-center of National Resource Center for Chinese Materia Medica, China Academy of Chinese Medical Science, 400065 Chongqing, China; 4 Novogene Bioinformatics Institute, Building 301, Zone A10 Jiuxianqiao North 13 Road, Chaoyang District, 100083 Beijing, China

**Keywords:** Genome, Secondary metabolism

## Abstract

*Coptis chinensis* Franch, a perennial herb, is mainly distributed in southeastern China. The rhizome of *C. chinensis* has been used as a traditional medicine for more than 2000 years in China and many other Asian countries. The pharmacological activities of *C. chinensis* have been validated by research. Here, we present a de novo high-quality genome of *C. chinensis* with a chromosome-level genome of ~958.20 Mb, a contig N50 of 1.58 Mb, and a scaffold N50 of 4.53 Mb. We found that the relatively large genome size of *C. chinensis* was caused by the amplification of long terminal repeat (LTR) retrotransposons. In addition, a whole-genome duplication event in ancestral Ranunculales was discovered. Comparative genomic analysis revealed that the tyrosine decarboxylase (*TYDC*) and (S)-norcoclaurine synthase (*NCS*) genes were expanded and that the aspartate aminotransferase gene (*ASP5*) was positively selected in the berberine metabolic pathway. Expression level and HPLC analyses showed that the berberine content was highest in the roots of *C. chinensis* in the third and fourth years. The chromosome-level reference genome of *C. chinensis* provides important genomic data for molecular-assisted breeding and active ingredient biosynthesis.

## Introduction

*Coptis chinensis* Franch or *Rhizoma Coptis* (2*n* = 2X = 18, Ranunculaceae) is a well-known medicinal plant that is mainly cultivated in Chongqing, Hubei, Hunan, Shanxi, and Guizhou, China. *C. chinensis* prefers to grow in 1200–1800 m high mountains and damp, shady and cold environments. In China, *C. chinensis* is called Huanglian, Weilian, Chuanlian or Jizhualian, and its rhizome has been used as a traditional Chinese medicine for more than 2000 years^1,2^. The search for plant-derived alkaloids beneficial to human health has received widespread attention. The pharmacological activities of *C. chinensis* rhizomes have been proven to play an important role in preventing or attenuating the development or progression of diseases^1,3–5^. Many studies have demonstrated the pharmacological activities of Huanglian rhizome preparations, especially the activity of novel alkaloids^1,3–5^. The bioactivities of *C. chinensis* alkaloids include broad-spectrum antimicrobial^5^, anticancer^6^, antioxidant^7,8^, antidiabetic^9,10^, attenuation depressive-like^11^, antiadipogenic^12^, and anti-inflammatory^13,14^ properties. Generally, *C. chinensis* mainly consists of six alkaloids, i.e., berberine, coptisine, palmatine, jatrorrhizine, epiberberine, and columbamine^15^. Among them, berberine (~7%), the dominant alkaloid, is known to have multiple beneficial physiological effects^16^. Despite the beneficial properties and commercial interest in *C. chinensis*, the distribution and number of wild *C. chinensis* are very limited.

At present, *C. chinensis* mostly originates from cultivation and is mainly produced in the Shizhu Tujia Autunomous County of Chongqing. *C. chinensis* has several special characteristics, including seed propagation, introduction to different regions, facultative outcrossing, self-flowering, and cross-pollination coexistence. Therefore, the cultivated populations of *C. chinensis* contain rich genetic resources that are diverse and highly heterozygous, and it is thus difficult to perform plant purification and breed selection.

To date, no species in the *Coptis* genus has a fully sequenced genome. High-quality genomes enable comparative analyses of genome architecture and the evolution of key traits for seed plants^17–19^. Despite the importance of *C. chinensis* as an ideal traditional medicine, genetic research on Huanglian is far from well developed, with no high-quality reference genomes. Here, we present the first high-quality genome assembly of *C. chinensis* using combined approaches, including PacBio (single-molecule Pacific Biosciences), Illumina HiSeq X, and Hi-C (genome-wide chromosome conformation capture) technology. This assembly contains 9 chromosomes (N50 = 4.53 Mb) totaling 958.20 Mb. The high-quality genomes of *C. chinensis* will allow us to uncover the genetic mechanisms behind special features such as the biosynthesis of alkaloids.

## Results

### Genome assembly and feature annotation

The genome size was estimated ~1046.91 Mb, the heterozygosity proportion was 0.77, and the repeat ratio was 73.96% based on k-mer analysis (Table S1, Fig. S1). A total of ~136.69 Gb of raw reads with an inserted 350 bp library (~130.57X), 118.36 Gb of PacBio data (~113.06X), 130.73 Gb of 10X genomics data (~124.87X), and 95.99 Gb of clean Hi-C reads (~91.69X) were used for assembly (Table S2), yielding a draft genome of 958.20 Mb with a contig N50 of 1.58 Mb and a scaffold N50 of 4.53 Mb, which is close to the estimates based on k-mer analysis (Table S3). Approximately 95.69% of the assembled scaffolds were anchored to nine pseudochromosomes based on Hi-C data (Fig. S2, Tables 1, S4).

**Table 1:** Statistics for assembly and annotation of the *Coptis chinensis* genome.

Characteristic	Number	Size
Assembly		
Estimated genome size		1046.91 Mb
Scaffolds	425	958.20 Mb
N50 of scaffolds	63	4.53 Mb
Longest scaffolds		19.44 Mb
Contigs	940	955.01 Mb
N50 of contigs	187	1.58 Mb
Longest contigs		8.97 Mb
Annotation		
Repetitive sequences	62.23%	596.28 Mb
Transposable element	60.75%	582.07 Mb
Protein-coding genes	34,109	
Mean transcript length		3846.49 bp
Mean coding sequence length		1031.05 bp
Noncoding RNAs	5424	615,322 bp

**Fig. 1:**
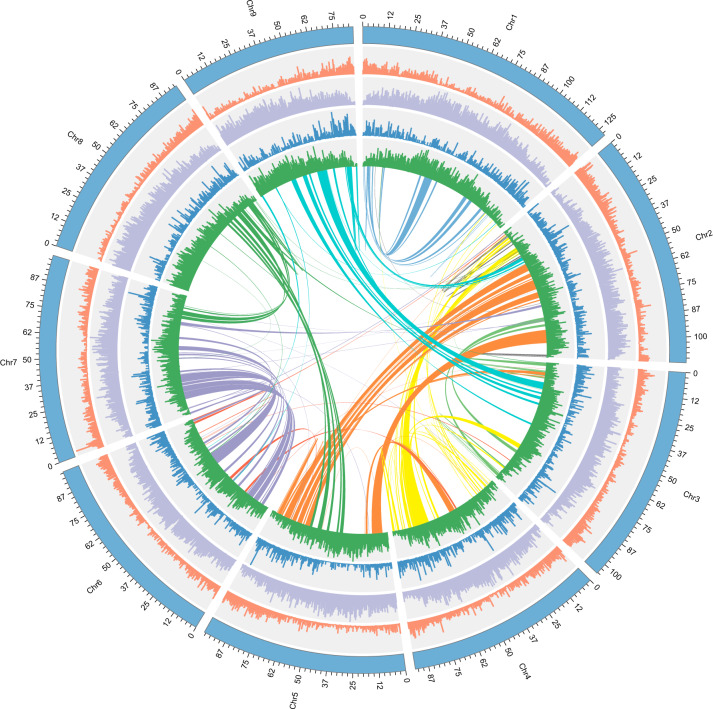
Genome features across 9 chromosomes. The outermost circle (blue) represents each chromosome of the genome. The bar charts of the second to fifth circles suggest gene density, LTR density, *Copia* and *Gypsy* density, respectively. The inner circular representation shows interchromosomal synteny. All sections were drawn based on window size = 300 kb and chromosome units = 500 kb

The mapping rate reached 96.79% when Illumina reads were aligned with our assembled genome, and the completeness of our genome assembly was evaluated by CEGMA^20^ and BUSCO assessment. The results showed that a total of 236 (95.16%) of 248 core eukaryotic genes (CEGs) and 1523 (94.3%) complete gene models among 1614 conserved genes from BUSCO assessment were identified (Tables S5, S6, S7), thus supporting that our genome assembly has a high quality and completeness. Additionally, LTR annotation was performed to estimate the assembly genome quality. Compared with *Aquilegia oxysepala* (16.7)^21^ and *Aquilegia coerulea* (12.6)^22^ in the family Ranunculaceae, a high LAI score of 17.86 showed that the assembly of *C. chinensis* yielded high sequence continuity and met the reference quality^23^. Overall, the assembled *C. chinensis* genome contains 595.32 Mb (62.13%) of repetitive sequences, most of which are transposable elements (581.03 Mb; 60.63%). Long terminal repeat (LTR) retrotransposons represent 49.84% of the genome, the largest percentage of repetitive elements (Tables S8, S9, Figs. 1, S3).

We identified 34,109 protein-coding genes (PCGs) in *C. chinensis* with an average of 4.70 exons per gene and an average coding sequence (CDS) length of 1031.26 bp. Of these, 33,898 (99.40%) could be functionally annotated in at least one database, i.e., NR, SwissProt, KEGG and InterPro, by homology. A total of 25,147 (73.70%) genes contained Pfam domains, and 30,773 (90.10%) genes were assigned GO terms. In addition, noncoding RNAs were also annotated in the *C. chinensis* genome, yielding 1,050 miRNAs, 996 tRNAs, 2,112 rRNAs, and 1265 snRNAs with average lengths of 131.56, 76.38, 128.96, and 116.68 bp, respectively (Tables 1, S10, S11, S12).

### Comparative genomic analysis and whole-genome duplication (WGD)

**Fig. 2:**
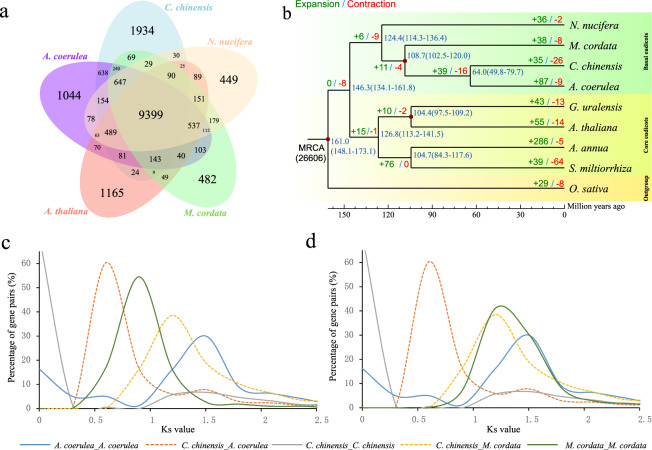
Comparison of gene families. **a** Venn diagrams displaying the number of gene families shared among five species. **b** Phylogenetic tree constructed by 817 single-copy genes. The divergence time is given in millions of years in blue. The gene families that expanded and contracted are given in green and red, respectively. **c**, **d** Percentage distribution of Ks for orthologous and paralogous gene pairs. The *x-*axis denotes the Ks value. The *y-*axis denotes the percentage of gene pairs. Rate distributions were not corrected (**c**); *Macleaya cordata* rates were corrected for *Coptis chinensis* (**d**)

To better understand the evolution of *C. chinensis*, we compared the sequence similarity of *C. chinensis* and 8 other species (*Aquilegia coerulea*, *Arabidopsis thaliana*, *Artemisia annua*, *Glycyrrhiza uralensis*, *Macleaya cordata*, *Nelumbo nucifera*, *Salvia miltiorrhiza*, and *Oryza sativa*) using OrthoMCL^24^. A total of 26,614 gene families were shared among *C. chinensis* and the eight other species, and 817 common single-copy orthologous gene families were identified according to sequence similarity. By comparing the gene families of *Coptis chinensis*, *Aquilegia coerulea*, *Arabidopsis thaliana*, *Macleaya cordata*, and *Nelumbo nucifera*, 1934 *Coptis chinensis*-specific gene families consisting of 5352 genes were detected (Fig. 2a). These genes were used to perform Kyoto Encyclopedia of Genes and Genomes (KEGG) functional annotation, and nine KEGG pathways were significantly enriched (cutoff < 0.05) (Table S13). The enriched phenylalanine, tyrosine, and tryptophan biosynthesis (map00400) could provide substrates for isoquinoline alkaloid biosynthesis, which probably has a potential contribution to the synthesis of benzylisoquinoline (BIAs).

A total of 817 single-copy orthologous gene families were used to perform phylogenetic analysis to confirm the relationship between *Coptis chinensis* and other species. According to phylogenetic tree results, *C. chinensis, A. coerulea*, *M. cordata*, and *N. nucifera* were clustered in the same branch, which belongs to the basal eudicot clade, and the result is consistent with their previously reported phylogenetic relationship. Further divergence time analysis showed that the Proteales order (*N. nucifera*) separated from the Ranunculales order (*A. coerulea*, *C. chinensis*, and *M. cordata*) ~124.4 (114.3–136.4) Mya ago, and *C. chinensis* separated from the same family species, *A. coerulea*, ~64.0 (49.8–79.7) Mya (Fig. 2b). We also detected the expanded or contracted gene families using CAFÉ (version 1.6). In total, 26,606 gene families were inferred in the most recent common ancestor (MRCA) of the nine species. Compared with the MRCA of *C. chinensis* and *A. coerulea*, 35 significantly expanded and 26 significantly contracted gene families were exhibited in *C. chinensis* (Fig. 2b). Genes in the 35 significantly expanded families were used to perform functional annotation based on the KEGG database, and ten KEGG pathways were significantly enriched (cutoff < 0.05) (Table S14). The enriched isoquinoline alkaloid biosynthesis (map00950) potentially demonstrates the genomic basis for the high BIA content in *C. chinensis*. All five genes enriched in this pathway belong to the tyrosine decarboxylase (*TYDC*) family, which is involved in the synthesis of premises (dopamine and 4-hydroxyphenylacetaldehyde) of BIAs. Thirty positively selected genes were identified in *C. chinensis*. According to the KEGG functional classification, isoquinoline alkaloid biosynthesis (map00950) was also enriched, and the enriched gene aspartate aminotransferase (*ASP5*) was also involved in the synthesis of premises (4-hydroxyphenylacetaldehyde) of BIAs (Table S15).

By calculating the Ks values of duplicate gene pairs, we observed a peak at Ks values of 1.5 (Fig. 2c, d), indicating that *C. chinensis* likely underwent a WGD event and that the peak of ~0 values of *C. chinensis* may be caused by tandem repeats. The peak value of orthologs between *C. chinensis* and *A. coerulea* (Ks = 0.6) was lower than the value of Ks = 1.2 between *C. chinensis* and *M. cordata* (Fig. 2c), implying that the speciation between *C. chinensis* and *A. coerulea* occurred later, and this result corresponds to the phylogenetic relationship. According to the distribution of Ks values, the WGD event of *M. cordata* occurred after speciation with *C. chinensis*, which suggests that *M. cordata* would share another WGD event with *C. chinensis* (Fig. 2c). However, there is no evidence to support that *M. cordata* had additional WGD events^25^. Such a result could be due to the relatively slow evolution of *M. cordata*. To confirm this, we determined Ks rates between *C. chinensis* and *N. nucifera*, finding that the mean Ks was larger than that between *M. cordata* and *N. nucifera*. Then, we adjusted the Ks distributions of *M. cordata* according to the ratio of the Ks value of the orthologous gene pairs between *C. chinensis-N. nucifera* and *M. cordata*-*N. nucifera* according to the method of Paterson et al.^26^ and Pei et al.^22^. The adjusted result showed that *C. chinensis*, *A. coerulea* and *M. cordata* occupied almost the same position, strongly implying that they shared a common WGD event (Fig. 2d).

### LTR retrotransposon expansion leads to a large genome size

Compared with the genomes of two closely related species, *M. cordata* (~378 Mb)^25^ and *A. coerulea* (~307 Mb)^27^, the genome size of *C. chinensis* (~958 Mb) is much larger. Transposable elements, especially LTRs, are essential for the formation of genome structure. The evolution of LTRs was investigated to explore the potential contribution to the enlargement of the *C. chinensis* genome. *C. chinensis* harbors the highest content of LTRs (~461.6 Mb), compared with the other two closely related species in Ranunculales: *M. cordata* (~104.8 Mb)^25^ and *A. coerulea* (~100.3 Mb)^27^. To trace the history of the expanded LTRs in *C. chinensis*, we identified LTRs and estimated insertion times of all intact LTRs in these species. A total of 23,433, 5231, and 6370 intact LTRs were identified in *C. chinensis*, *M. cordata*, and *A. coerulea*, respectively. In the genomes of *M. cordata*, no significant proliferation of LTRs was observed. In the genome of *A. coerulea*, there were obviously more LTR insertions than in *M. cordata* in the last 2 Mya. LTRs continuously and substantially accumulated only in the genome of *C. chinensis* in the last 10 Mya, which showed a relatively longer expansion period than in the other two species (Fig. S4).

**Fig. 3:**
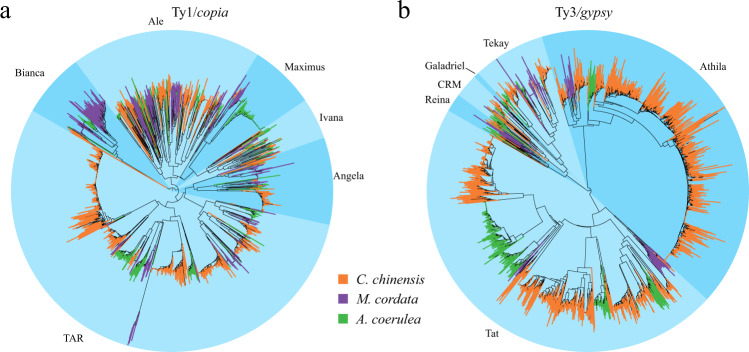
Phylogenetic analysis of LTRs in the *C. chinensis* genome. The neighbor-joining phylogenetic trees based on Ty1/*copia* (**a**) and Ty3/*gypsy* (**b**). Major lineages are named, and the proportion is indicated

Approximately 81.67% of the intact LTRs in the *C. chinensis* genome had at least one protein domain, of which the vast majority were Ty1/*copia* and Ty3/*gypsy*, accounting for 19.40% and 80.53%, respectively, and their corresponding total lengths were 23.96 and 187.96 M. The proportion of Ty3/*gypsy* elements in the *C. chinensis* genome was significantly higher than that in *M. cordata* (47.41%) and *A. coerulea* (52.42%). In addition, the total length of Ty3/*gypsy* elements in *the C. chinensis* genome was 12.51 times longer than that in *M. cordata* and 8.77 times longer than that in *A. coerulea*. Ty1/*copia* elements also expanded in *C. chinensis*, with total lengths 1.83 and 1.73 times those of *M. cordata* and *A. coerulea*, respectively (Table S16). The evolutionary relationships of individual Ty1/*copia* and Ty3/*gypsy* LTR superfamilies were studied in these three species. All Ty3/*gypsy* elements from these three species were grouped into six major evolutionary clades, i.e., Angela, Ale, Bianca, Ivana, Maximus, and TAR (Table S17). The Ty3/*gypsy* elements of *M. cordata* and *A. coerulea* were separated into six major evolutionary lineages: Tekay, Galadriel, CRM, Reina, Athila, and Tat. However, the Ty1/*copia* elements of *C. chinensis* were assigned to five distinct lineages, of which the Galadriel lineage was not found (Table S18). To understand the amplification of individual lineages, we calculated the copy numbers and constructed phylogenetic trees of Ty1/*copia* and Ty3/*gypsy*. There was no significant expansion of Ty1/*copia* and Ty3/*gypsy* in *M. cordata*. In *A. coerulea*, the Tat of Ty3/gypsy showed significant expansion, which accounted for 78.46% of the total (Table S18, Fig. 3b), which was consistent with more LTR insertions in *A. coerulea* over the last 2 Mya. More LTR insertions resulted in a higher proportion of LTRs in *A. coerulea* (32.67%) than in *M. cordata* (27.72%). In *C. chinensis*, the significantly expanded lineage of the Ty1/*copia* superfamily was TAR, which accounted for 69.09% (Table S17, Fig. 3a), and the Athila and Tat of Ty3/*gypsy* also showed significant expansion, which accounted for 40.17% and 53.02%, respectively (Table S17, Fig. 3b). The three significantly expanded lineages may be drivers of the expanded genome of *C. chinensis* (Tables S16–18, Fig. 3).

### Benzylisoquinoline alkaloid biosynthesis-associated genes

In total, 63 candidate genes were identified based on 14 previously reported genes involved in BIA biosynthesis, i.e., tyrosine decarboxylase (*TYDC*), tyrosine/tyramine 3-hydroxylase/tyrosine 3-monooxygenase (*3OHase*), L-tyrosine aminotransferase (*TyrAT*), (S)-norcoclaurine synthase (*NCS*), (RS)-norcoclaurine 6-O-methyltransferase (*6OMT*), (S)-coclaurine N-methyltransferase (*CNMT*), (S)-N-methylcoclaurine 3′-hydroxylase/N-methylcoclaurine 3′-monooxygenase (*NMCH*), 3′-hydroxy-N-methyl-(S)-coclaurine 4′-O-methyltransferase (*4*′*OMT*), berberine bridge enzyme (reticuline oxidase) (*BBE*), (S)-scoulerine 9-O-methyltransferase (*SOMT*), (S)-canadine synthase (*CAS*), (S)-tetrahydroprotoberberine oxidase (*STOX*), (S)-corytuberine synthase (*CTS*), columbamine O-methyltransferase (*CoOMT*), and reticuline N-methyltransferase (*RNMT*) ^28^. To clarify the specific evolution of genes involved in BIA synthesis in Ranunculales, gene orthologs were also identified in *M. cordata*, *A. coerulea* and *A. thaliana*. (S)-Reticuline is an intermediate required for the synthesis of many BIAs. Compared with *Arabidopsis thaliana*, four genes (*NCS*, *6OMT*, *NMCH*, and *4*′*OMT*) involved in the pathways from L-tyrosine to (s)-reticuline could only be found in three Ranunculales plants (Table S19), and another key gene, *CNMT*, was also significantly expanded in Ranunculales plants. Comparative analysis with *Aquilegia coerulea* and *Macleaya cordata* showed that the key gene *CoOMT* responsible for catalyzing columbamine into palmatine expanded in *C. chinensis*, and the key gene *NCS* involved in the synthesis of (S)-norcoclaurine, a precursor to BIAs, also expanded in *C. chinensis* (Table S19).

To better understand the molecular mechanisms and regulatory processes of BIAs, we investigated the expression patterns of genes potentially related to BIA biosynthesis from five tissues (flowers, seeds, roots, fibrous roots, and leaves) at the same stage and roots from 3 to 6 years. According to previous research, berberine, coptisine and jatrorrhizine were significantly more abundant in the roots than in the rhizomes and leaves of *C. chinensis*^28^. In our study, the expression level analysis of different tissues showed that the genes *STOX* and *CoOMT*, acting on the final catalytic for the synthesis of predominant BIAs (berberine, coptisine, and palmatine), were significantly overexpressed in fibrous roots and roots compared with other tissues. In addition, the expression levels of the key genes *BBE* and *4*′*OMT* were also higher in roots. Many genes, those with multiple copies, exhibited an obvious divergence in expression pattern among copies from different tissues, such as *NCS*, *NMCH*, and *CNMT* (Fig. S5). As the BIA content was the highest in the roots of *C. chinensis*, we further analyzed the expression levels of roots in different years. Most of the candidate genes related to BIA synthesis were most highly expressed in the third year, followed by the fourth year, and were significantly decreased in the fifth and sixth years (Figs. S6, 4a), which suggests that the key period for BIA synthesis in *C. chinensis* is the third and fourth years.

**Fig. 4:**
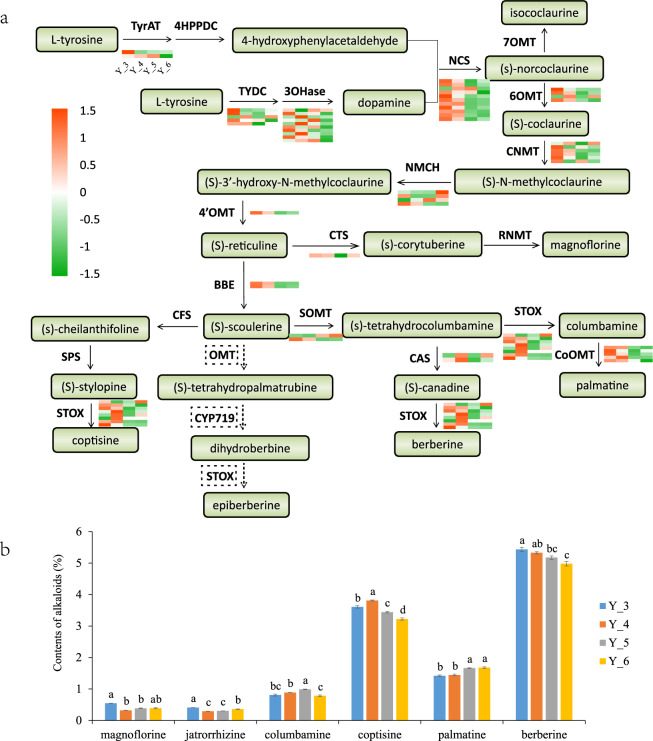
BIA biosynthesis in *C. chinensis*. **a** Gene expression in BIA metabolism pathways. In each heatmap, the columns from left to right represent the expression level of the roots of *C. chinensis* from 3 to 6 years. **b** The magnoflorine, jatrorrhizine, columbamine, coptisine, palmatine, and berberine contents in *C. chinensis* roots from 3 to 6 years based on HPLC. Values are means ± SE. Values with the same letter within each BIA type are not significantly different

To verify that the candidate genes were closely related to the content of BIAs, high-performance liquid chromatography (HPLC) analysis was used to detect the presence of magnoflorine, jatrorrhizine, columbamine, berberine, coptisine, and palmatine in the roots of *C. chinensis* from 3 to 6 years. In general, the highest proportion of BIAs in the roots of *C. chinensis* was berberine, followed by coptisine and palmatine. The content of predominant BIAs (berberine and coptisine) in *C. chinensis* reached the highest level in the fourth year and then decreased, which indicates that BIAs in *C. chinensis* accumulated until the fourth year (Fig. 4b). Correlation coefficient analysis of gene expression patterns and various alkaloid contents was used to further validate candidate genes. The results showed that most of the key genes were highly correlated with the predominant components (berberine and palmatine), with *R*^2^ > 0.8, such as *NCS*, *6OMT*, *CNMT*, *BBE*, and *STOX* (Fig. S7). Coptisine is also the predominant component of BIAs in *C. chinensis*; however, *CFS* and *SPS* genes related to the synthesis of coptisine have not been identified, which may be because other enzymes of the CYP719 family have similar functions or there are other bypass pathways for the synthesis of (S)-stylopine. In addition, recently duplicated gene blocks (Ks < 0.3) were used to investigate the effects on BIA synthesis in *C. chinensis*. We found that *3OHase 6OMT* and *CNMT* were in the recently duplicated blocks (Fig. S8).

## Discussion

A high-quality, chromosome-level genome provides a key resource to study how biosynthetic pathways evolved^21^. To date, only genomes of *Aquilegia oxysepala*^29^ and *Aquilegia coerulea*^27^ from the family Ranunculaceae have been reported. In our study, we obtained a high-quality genome of cultivated *C. chinensis* using a combined approach. The final chromosome-level genome assembly is ~958.20 Mb, which is close to the assessed genome size (1.06 Gb), and similar to the previous version of the *C. chinensis* genome^30^. The assembled genome has a high quality and completeness, with a contig N50 of 1.58 Mb and a scaffold N50 of 4.53 Mb; in total, 34,109 PCGs were predicted. This is the first chromosome-level reference genome of *C. chinensis*, which provides important genomic data for *C. chinensis* studies.

The first genome in the *Coptis* genus facilitates further research on fundamental comparative and genome evolution. Among *Coptis*-expanded and positive genes, isoquinoline alkaloid biosynthesis activity is one of the more highly enriched KEGG pathways, including the genes (*TyrAT* and *TYDC*) related to the conversion from L-tyrosine to 4-hydroxyphenylacetaldehyde and dopamine, suggesting that they are part of the genomic basis for the high content of isoquinoline, including berberine, in *C. chinensis*. Comparison of the *C. chinensis*, *A. coerulea* and *M. cordata* genomes supports the hypothesis that basal eudicot plants lack the paleohexaploidy event and that the ancestor of Ranunculales shared a common WGD^25,29^ (Fig. 2d). The distribution of Ks values of duplicated gene pairs in corresponding blocks suggests that the divergence of *C. chinensis*—*A. coerulea* occurred later than that of *C. chinensis*—*M. cordata* (Fig. 2d). *C. chinensis* possesses a significantly enlarged genome compared with closely related species in the Ranunculaceae family. A positive correlation between the content of LTRs and genome size was previously reported for maize^31^, pineapple^32^, and tea tree^33^. In our study, the repeat-sequence contents of TAR, Athila and Tat of LTR lineages were drivers of the enlarged *C. chinensis* genome.

Furthermore, we also detected genes related to BIAs in the *C. chinensis* genome, and the contents of BIAs were detected by HPLC. The biosynthesis of BIAs starts with the transformation from L-tyrosine to 4-hydroxyphenylacetaldehyde and dopamine, by which (S)-norcoclaurine is synthesized under the catalysis of *NCS*. Three methyltransferases (*6OMT*, *CNMT*, and *4*′*OMT*) and one cytochrome P450 (*NMCH*) are associated with the transformation from (S)-norcoclaurine to (S)-reticuline, which is necessary for the synthesis of multiple BIAs. The *NCS* genes involved in this process are significantly expanded in *Coptis chinensis*, which may provide sufficient substrates for downstream synthesis. Some key genes related to BIA synthesis, such as *STOX*, *CoOMT*, *BBE*, and *4*′*OMT*, have high expression levels in roots and fibrous roots. This suggests that roots are the main organ for the synthesis of BIAs, which is consistent with a previous study^28^. Consistent with the HPLC analysis results, most candidate genes related to BIA synthesis were highly expressed in the third and fourth years, indicating that these candidate genes contributed to the synthesis of BIAs and provide basic data for the selection of the best medicinal period for *C. chinensis*. Three key genes for the synthesis of the intermediate (S)-reticuline (*3OHase 6OMT* and *CNMT*) were recently duplicated (Fig. S8), suggesting that recent duplications may have a positive effect on the biosynthesis of BIAs in *Coptis chinensis*.

For *Coptis chinensis*, an important medicinal plant, the reported chromosome-level genome lays a foundation for the analysis of comparative genomes and will contribute to molecular breeding, as well as the elucidation of BIA metabolic pathways.

## Materials and methods

### Plant materials

Diploid *C. chinensis* was originally collected from the Chongqing Academy of Chinese Materia Medica, Chongqing, China (29.99°N, 108.44°E, 1667 m asl). Young leaves in a slowly growing period from a 5-year-old individual were used for genome sequencing and assembly.

### Library construction and genome sequencing

Genomic DNA was extracted from fresh leaf tissues of *C. chinensis* using the plant Genomic DNA Kit (Tiangen, Beijing, PR China) according to the manufacturer’s instructions. Libraries of 350 bp were constructed and sequenced using the Illumina HiSeq platform (Illumina, San Diego, CA).

Low-quality reads and sequences with adapters were removed from the raw data. The following reads were removed from the raw data:

(1) Reads containing adapters.

(2) Paired reads containing unidentified nucleotides (N) >10% of the total bases of single reads.

(3) Paired reads containing more than 20% low-quality bases (Phred <5) of the total length of single reads.

Libraries of 20 kb for single-molecule real time require at least 10 μg of sheared DNA. The libraries were sequenced using the PacBio Sequel platform. Hi-C libraries were also enriched, sheared, and sequenced using Illumina HiSeq. Five tissues (leaf, seed, flower, root, and fibrous root) from the same individual were collected for RNA sequencing.

### De novo genome assembly and genome quality assessment

K-mer frequency analysis was performed^34^ to estimate the *C. chinensis* genome size, heterozygosity and repeat content. A 17-mer frequency was generated from high-quality 250 and 450 bp paired-end reads. The distribution of 17-mers following a Poisson’s distribution can reflect the characteristics of the genome. The long subreads generated by PacBio were assembled using the following steps. First, error correction of the raw subreads was performed to obtain preassembled reads, which generally have a very high accuracy (up to 99.999%). Then, Falcon^35^ software was applied to assemble the preassembled reads using the overlap-layout-consensus (DBG2OLC) method. The production of primary contigs (p-contigs) was performed using FALCON-Unzip^35^, and then Quiver^36^ was used for polishing. Finally, Pilon^19^ was used to perform error correction of p-contigs, and FragScaff^37^ was used to generate scaffolds. Finally, the anchorage of the genome assembly onto chromosomes was performed by the LACHESIS pipeline^38^.

To assess the quality of the assemblies (accuracy and completeness), Illumina clean reads were mapped to our assembly using BWA^39^. In addition, CEGMA (Core Eukaryotic Genes Mapping Approach)^20^, BUSCO (Benchmarking Universal Single-Copy Orthologs, embryophyta_odb10 database)^40^, and the LTR Assembly Index (LAI)^23^ were used to assess the genome.

### Annotation of repetitive elements

The repetitive elements of both assemblies were identified using combined strategies, homology-based and de novo prediction. RepeatModeler (http://www.repeatmasker.org/RepeatModeler.html), RepeatScout^41^ (http://www.repeatmasker.org/), and LTR-Finder^42^ (http://tlife.fudan.edu.cn/ltr_finder) were applied to ab initio repeat element library construction with default parameters, and RepeatProteinMask and RepeatMasker (http://www.repeatmasker.org) were used to identify repeats. The homologous annotations of repetitive elements were also performed using RepeatProteinMask and RepeatMasker (http://www.repeatmasker.org) by alignment to the consensus sequences in the Repbase library^43^.

### Prediction and functional annotation of genes

Combined strategies, including ab initio, homology-based, and transcript-assisted strategies, were applied to identify genes in the *Coptis chinensis* assemblies. EvidenceModeler^44^ (EVM, http://evidencemodeler.sourceforge.net) was applied to integrate the annotation results of all the methods to produce the final gene models. The software PASA^45^ (https://github.com/PASApipeline/PASApipeline/wiki) was then applied for the identification of alternative splicing variations and untranslated sequences (UTRs) of genes. The domains and motifs of the gene models were identified by InterProScan^46^. Gene function annotations were performed by aligning them to the InterPro, NR, SwissProt, and KEGG databases.

### Synteny analyses

All genes were compared in pairs by BLASTP (*E* value < 10^−5^) to obtain orthologous gene pairs. Collinear blocks containing fewer than five orthologous gene pairs were filtered out by MCScanX^47^. The collinearity information of the gene set and other genomic features in the *C. chinensis* genome were visualized by Circos (v0.69)^48^.

### Gene family clustering

The protein sequences of *Coptis chinensis* and 7 other plant species (*Aquilegia coerulea*, *Arabidopsis thaliana*, *Artemisia annua*, *Glycyrrhiza uralensis*, *Macleaya cordata*, *Nelumbo nucifera*, and *Salvia miltiorrhiza*) with the outgroup species *Oryza sativa* were used for gene family clustering. Genes encoding no more than 50 amino acids were removed. For all the genes of the nine species, when there were multiple transcripts in the same gene, only the longest was used for subsequent analysis. The similarity among the remaining genes from all species was obtained by comparing pairs using BLASTP with a cutoff *E* value of 1e−5. OrthoMCL (http://orthomcl.org/orthomcl/) was used to identify paralogous and orthologous protein sequences among the nine species with an inflation parameter of 1.5.

### Phylogenetic analysis

The coding sequences (CDSs) were extracted from single-copy gene families shared by nine species and were aligned by MUSCLE according to the alignments of the protein sequences. Each family was concatenated into a superalignment matrix. RAxML (http://sco.h-its.org/exelixis/web/software/raxml/index.html) was applied to construct the maximum likelihood phylogeny using the GTRGAMMA model with *Oryza sativa* as an outgroup, and the bootstrap value was set to 100.

### Estimation of divergence time

The MCMCtree program of PAML (http://abacus.gene.ucl.ac.uk/software/paml.html) was implemented to estimate the differentiation time according to the maximum likelihood phylogeny tree with the following settings: 100,000 iterations were discarded, sampling number = 100,000, and sampling frequency = 2. The times of divergence between monocotyledons and dicotyledons (148–173 Mya) *Aquilegia coerulea* and *Macleaya cordata* (103–122 Mya), *Glycyrrhiza uralensis* and *Arabidopsis thaliana* (97–109 Mya), which refer to the TimeTree database (http://www.timetree.org/) and previous studies^25,49^, were used for recalibration.

### Gene family analysis

The gene families of each species that had undergone expansion or contraction were identified by comparison with the number of genes contained in each ancestral gene family using CAFE software (version 1.6). All gene family changes along each lineage of the phylogenetic tree were studied by the random birth and death model. The extrapolation of changes in the gene family size and the direction of changes in the phylogeny were ascertained by the probabilistic graphical model.

### Positive selection gene

The protein sequences of single-copy gene families from *Coptis chinensis*, *Arabidopsis thaliana*, *Aquilegia coerulea*, *Glycyrrhiza uralensis*, *Salvia miltiorrhiza*, *Artemisia annua*, *Macleaya cordata*, and *Nelumbo nucifera* were extracted and aligned by MUSCLE (version 3.7). Then, Gblocks (version 0.91b85) was used to extract the conserved sites of the protein sequence alignments. The Codeml program of PAML software (version 4.7) was applied to estimate the evolutionary rate of each branch with a free-ratio model. Then, we detected the positive selection signals on genes of each family using the branch-site model with *C. chinensis* as the foreground branch and the other seven species as the background branch. The likelihood ratio test was used to detect candidates that underwent positive selection with a cutoff *p* value of 0.05.

### Whole-genome duplication analysis

The protein sequences from the *Coptis chinensis*, *Aquilegia coerulea*, *Macleaya cordata*, and *Nelumbo nucifera* genomes were used to analyze WGD events. The BLASTP program of blastall (version 2.2.26) was used to perform a homolog search with a cutoff *E* value of 10^−5^, and MCScanX was applied to obtain syntenic blocks with default parameters. Finally, the gene pairs located in syntenic blocks were aligned using MUSCLE, and Ks (synonymous substitution rate) values were calculated using the YN algorithm to detect putative WGD events^50^.

### High-performance liquid chromatography analysis

Dry powder samples (0.2 g) were extracted with 50 ml hydrochloric acid ethanol mixed liquor (1:100, v/v) for 30 min and sonicated for 30 min. Determination of the major bioactive components of *C. chinensis* was performed on an Agilent 1260 HPLC system (Agilent Technologies, Santa Clara, Calcium, USA) with an Agilent ZORBAX SB-C 18 column (250 mm × 4.6 mm, 5 mm, Agilent Technologies) at a column temperature of 30 °C and a flow rate of 1 mL/min. The mobile phase was acetonitrile—0.05 mol/L potassium dihydrogen phosphate solution (50:50, v/v), containing 0.1% sodium dodecyl sulfate, separated by equal-diameter elution. The detection wavelength was 345 nm. The contents of berberine, palmatine, cannine, coptidine, magnoline, and columbine were calculated by standard curves^28,51^. One-way analysis of variance was used to compare various BIA contents among roots in different years. If the variances were significantly different, Tamhane’s T2 test was used to perform post hoc analyses; otherwise, a least significant difference test was used. The results were considered significant at *p* < 0.05.

### Accession numbers

All raw and processed sequencing data generated in this study have been uploaded to the NCBI BioProject database under accession number PRJNA649082. The raw genome sequencing data obtained by the Illumina and PacBio platforms were uploaded to the NCBI BioSample database under accession number SAMN15658056. The raw sequencing data of the transcriptome have been uploaded to the NCBI BioSample database under accession number SAMN15658057.

## Supplementary information

Supplementary tables 1–19

Supplemental Figure 1

Supplemental Figure 2

Supplemental Figure 3

Supplemental Figure 4

Supplemental Figure 5

Supplemental Figure 6

Supplemental Figure 7

Supplemental Figure 8
