# Supplementary material for: The chromosome-level reference genome of Coptischinensis provides insights into genomic evolution and berberine biosynthesis
Source: Hortic Res. 2021 Jun 1;8:121. doi: 10.1038/s41438-021-00559-2 (PMC8166882; doi:10.1038/s41438-021-00559-2)
Supplement: Supplementary file 1 — Supplementary tables 1–19 [file 41438_2021_559_MOESM1_ESM.pdf]

Supplemental Table 1. 17 K-mer statistics

K-mer	K-mer number	K-mer Depth	Genome Size (Mbp)	Revised Genome Size	Heterozygous Ratio (%)	Repeat (%)
17	103,815,432,824	98	1059.34	1046.91	0.77	73.96

Supplemental Table 2. The sequencing data of *Coptis chinensis*

Pair-end libraries	Insert size	Total data (G)	Read length (bp)	Sequence coverage (X)
Illumina reads	350bp	136.69	150	130.57
PacBio reads	—	118.36	—	113.06
10X Genomics	—	130.73	150	124.87
Hic		95.99		91.69
Total	—	481.77	—	460.19

Supplemental Table 3. The de novo assembly results of *Coptis chinensis* .

Characteristic	Length		Number	
	Contig** (bp)	Scaffold (bp)	Contig**	Scaffold
Total	955006981	958200615	940	425
Max	8967247	19442731	-	-
Number>=2000	-	-	939	424
N50	1581007	4529715	187	63
N60	1239416	3361228	255	88
N70	1,028,899	2389863	339	122
N80	811539	1837752	444	168
N90	540118	1126120	587	233

** Contig after scaffolding

Supplemental Table 4. The length of psedo-chrmosomes in genome of *Coptis chinensis*.

Chrsomosome ID	Length
Chr1	128756622
Chr2	112244699
Chr3	105526292
Chr4	93906356
Chr5	98305372
Chr6	98076476
Chr7	99670480
Chr8	95914542
Chr9	84534767
Total	916,935,606(95.69%)

**Supplemental Table 5. Coverage statistics of genome of *Coptis chinensis***		
Characteristic		Percentage
Reads	Mapping rate (%)	96.79
	Average sequencing depth	114.76
	Coverage (%)	99.85
Genome	Coverage at least 4X(%)	99.77
	Coverage at least 10X(%)	99.65
	Coverage at least 20X(%)	99.4

Supplemental Table 6. CEGMA evaluation results.

Complete		Complete + partial	
# Prots	% Completeness	# Prots	% Completeness
227	91.53	236	95.16

Complete: core genes that were completely assembled with identity greater than 0.7.

Partial: core genes that were assembled but not complete.

Prots: the number of core genes.

% Completeness: percentage of assembled core genes to core genes.

Supplemental Table 7. BUSCO assessment of genome of *Coptis chinensis*.

Species	BUSCO notation assessment results
Coptis chinensis	C:94.3%[S:86.7%,D:7.6%],F:1.3%,M:4.4%,n:1614

#C: Complete Single-Copy BUSCOs; S：Complete and single-copy BUSCOs; D: CompleteDuplicated BUSCOs; F: Fragmented BUSCOs; M: Missing BUSCOs; n: Total BUSCO groupssearched

Supplemental Table 8. The statistics of repeat sequences in genome of *Coptis chinensis*

Type	Repeat Size(bp)	% of genome
Trf	45780607	4.78
Repeatmasker	540011062	56.35
Proteinmask	199014763	20.77
Total	595318868	62.13

Supplemental Table 9. The statistics of tandem repeat elements in genome of *Coptis chinensis*

Type	Denovo+Repbase Length(bp)	% in Genome	TE proteins Length(bp)	% in Genome	Combined TEs Length(bp)	% in Genome
DNA	43488403	4.54	27177795	2.84	59437400	6.2
LINE	21386316	2.23	30348024	3.17	41692022	4.35
SINE	325789	0.03	0	0	325789	0.03
LTR	461603807	48.17	142112943	14.83	476662657	49.74
Unknown	19987462	2.09	0	0	19987462	2.09
Total	540011062	56.35	199014763	20.77	581034160	60.63

Supplemental Table 10. The statistics of gene structure in genome of *Coptis chinensis*

Methods	Gene set	Number	Average transcript length(bp)	Average CDS length(bp)	Average exons per gene	Average exon length(bp)	Average intron length(bp)
De novo	Augustus	46582	2822.45	927.75	4	231.83	631.16
	GlimmerHMM	90383	9241.9	561.71	2.87	195.45	4632.23
	SNAP	68186	5601.86	671.51	4.11	163.25	1583.59
	Geneid	77846	4591.44	693.76	4.12	168.31	1248.45
	Genscan	49458	12433.01	1089.48	6	181.67	2270.01
Homolog	Arabidopsis thaliana	74185	1452.66	696.1	2.24	310.41	608.91
	Beta vulgaris	55341	2296.58	1040.29	2.84	366.71	683.96
	Macleaya cordata	71722	2018.9	795.92	2.51	317	809.5
	Nelumbo nucifera	32996	3597.18	1228.59	3.62	339.38	904.02
	Oryza sativa	55337	2049.74	1040.22	2.53	411.62	661.05
	Solanum lycopersicum	82108	1526.25	784.51	2.2	357.16	619.91
	Vitis vinifera	48961	2138.13	854.95	2.95	289.89	658.31
RNAseq	PASA	20154	4041.4	1281.71	5.21	245.99	655.45
	Cufflinks	60547	7089.25	1976.87	5.98	330.78	1027.31
EVM		53781	3135.82	873.55	3.98	219.5	759.23
Pasa-update*		53736	3106.59	877.43	3.99	220.02	746.06
Final set*		34109	3830.72	1030.26	4.7	219.39	757.71

Supplemental Table 11. The statistics of annotated gene in genome of *Coptis chinensis*

characters	Number	Percent(%)
Total	34109	-
Swissprot	26818	78.6
Nr	32481	95.2
KEGG	25318	74.2
InterPro	33639	98.6
GO	30737	90.1
Pfam	25147	73.7
Annotated	33898	99.4
Unannotated	211	0.6

Supplemental Table 12. The statistics of non-coding RNA in genome *Coptis chinensis*.

Type		Copy(w*)	Average length(bp)	Total length(bp)	% of genome
miRNA		1050	113.56	119237	0.012443
tRNA		996	76.38	76071	0.007939
rRNA	rRNA	2112	128.96	272354	0.028422
	18S	49	860.43	42161	0.0044
	28S	70	141.93	9935	0.001037
	5.8S	20	156.3	3126	0.000326
	5S	1973	110.05	217132	0.022659
snRNA	snRNA	1265	116.68	147598	0.015403
	CD-box	844	104.15	87903	0.009173
	HACA-box	88	128.85	11339	0.001183
	splicing	333	145.21	48356	0.005046

Supplemental Table 13. The KEGG enrichment result of specific gene families in *Coptis chinensis*

MapID	MapTitle	Pvalue	AdjustedPv	Number	EnrichDirect
map00360	Phenylalanine metabolism	2.84E-06	0.000740709	36	Over
map00910	Nitrogen metabolism	8.25E-06	0.000740709	25	Over
map00960	Tropane, piperidine and pyridine alkaloid biosynthes	1.60E-05	0.000740709	28	Over
map03010	Ribosome	0.00022	0.006307714	155	Over
map00400	Phenylalanine, tyrosine and tryptophan biosynthesis	0.000299	0.007853052	33	Over
map00270	Cysteine and methionine metabolism	0.001072	0.020726313	56	Over
map00660	C5-Branched dibasic acid metabolism	0.001624	0.028420523	12	Over
map03420	Nucleotide excision repair	0.002824	0.041185144	50	Over
map04710	Circadian rhythm	0.002876	0.041185144	35	Over

Supplemental Table 14. The KEGG enrichment result of expanded gene families in *Coptis chinensis*

MapID	MapTitle	Pvalue	AdjustedPv	Gene_Number
map00480	Glutathione metabolism	1.29E-29	4.92E-28	33
map00670	One carbon pool by folate	1.12E-14	2.84E-13	13
map00561	Glycerolipid metabolism	2.83E-13	5.39E-12	22
map04139	Regulation of mitophagy - yeast	0.000208107	0.002636018	6
map03440	Homologous recombination	0.000647791	0.007033159	7
map04141	Protein processing in endoplasmic reticulum	0.001526928	0.012894056	14
map00450	Selenocompound metabolism	0.00198186	0.015062137	4
map03460	Fanconi anemia pathway	0.005716913	0.039498671	7
map00950	Isoquinoline alkaloid biosynthesis	0.008310742	0.045788948	5
map00564	Glycerophospholipid metabolism	0.008434806	0.045788948	8

Supplemental Table 15. The KEGG enrichment result of positively selected genes in *Coptis chinensis*

MapID	MapTitle	Pvalue	AdjustedPv	Gene_Number
map00232	Caffeine metabolism	0.008087147	0.24633729	1
map03015	mRNA surveillance pathway	0.035409836	0.24633729	2
map00196	Photosynthesis - antenna proteins	0.053411026	0.24633729	1
map00983	Drug metabolism - other enzymes	0.059177582	0.24633729	1
map00220	Arginine biosynthesis	0.071558996	0.24633729	1
map00960	Tropane, piperidine and pyridine alkaloid biosynthesis	0.081916094	0.24633729	1
map03013	RNA transport	0.092889543	0.24633729	2
map00250	Alanine, aspartate and glutamate metabolism	0.10413861	0.24633729	1
map00360	Phenylalanine metabolism	0.107792969	0.24633729	1
map00400	Phenylalanine, tyrosine and tryptophan biosynthesis	0.112341265	0.24633729	1
map03430	Mismatch repair	0.11415449	0.24633729	1
map00330	Arginine and proline metabolism	0.120473469	0.24633729	1
map00950	Isoquinoline alkaloid biosynthesis	0.120473469	0.24633729	1
map04530	Tight junction	0.123168645	0.24633729	1
map01210	2-Oxocarboxylic acid metabolism	0.133872116	0.252869552	1
map00350	Tyrosine metabolism	0.151439471	0.268128605	1
map00860	Porphyrin and chlorophyll metabolism	0.170377483	0.268128605	1
map04146	Peroxisome	0.177165242	0.268128605	1
map00270	Cysteine and methionine metabolism	0.216003497	0.284576648	1
map00520	Amino sugar and nucleotide sugar metabolism	0.24924944	0.313869666	1
map00240	Pyrimidine metabolism	0.314668848	0.378881647	1
map03018	RNA degradation	0.323163758	0.378881647	1
map00230	Purine metabolism	0.360183687	0.408208179	1
map01230	Biosynthesis of amino acids	0.441081056	0.463583318	1
map04144	Endocytosis	0.441081056	0.463583318	1
map03040	Spliceosome	0.459037892	0.463583318	1
map04141	Protein processing in endoplasmic reticulum	0.463583318	0.463583318	1

Supplemental Tables 16. Summary of intact LTRs with at least one protein-coding gene in *C. chinensis*.

	Copies			Length (Mb)		
Species	Gypsy	Copia	Others	Gypsy	Copia	Others
C. chinensis	15379	3704	14	187.96	23.96	0.14
M. cordata	1638	1811	6	15.02	13.09	0.03
A. coerulea	2217	1991	21	21.43	13.83	0.24

Supplemental Tables 17. The proportion (%) of lineages in Ty1/*copia* super family.

	Angela	Ale	Bianca	Ivana	Maximus	TAR
C. chinensis	8.57	18.00	0.87	3.17	0	69.09
M. cordata	14.75	23.01	23.50	1.46	6.32	30.96
A. coerulea	3.05	17.97	4.07	9.66	23.90	41.36

Supplemental Tables 18. The proportion (%) of lineages in Ty3/*gypsy* super family.

	Tekay	Tat	CRM	Athila	Galadriel	Reina
C. chinensis	4.71	40.17	0.96	53.02	0	1.13
M. cordata	36.79	26.68	9.59	17.62	1.81	7.51
A. coerulea	6.34	78.46	5.07	5.56	2.05	2.53

Supplemental Tables 19. The main identified BIA pathway biosynthetic enzyme genes from *C. chinensis* , *A. coerulea* , *M. cordata* and *A. thaliana* .

Genes	*C. chinensis*	*A. coerulea*	*M. cordata*	*A. thaliana*
3OHase	10	9	9	14
4'OMT	1	1	1	0
6OMT	4	3	2	0
BBE	2	5	2	14
CAS	2	8	1	0
CNMT	6	3	6	1
CoOMT	5	2	0	0
CTS	1	1	0	0
NCS	11	5	3	0
NMCH	4	3	1	0
SOMT	2	2	1	0
STOX	8	13	32	2
TryAT	2	3	4	6
TYDC	5	3	14	2
