# Supplementary figures and images for: The chromosome-level reference genome of Coptischinensis provides insights into genomic evolution and berberine biosynthesis
Source: Hortic Res. 2021 Jun 1;8:121. doi: 10.1038/s41438-021-00559-2 (PMC8166882; doi:10.1038/s41438-021-00559-2)

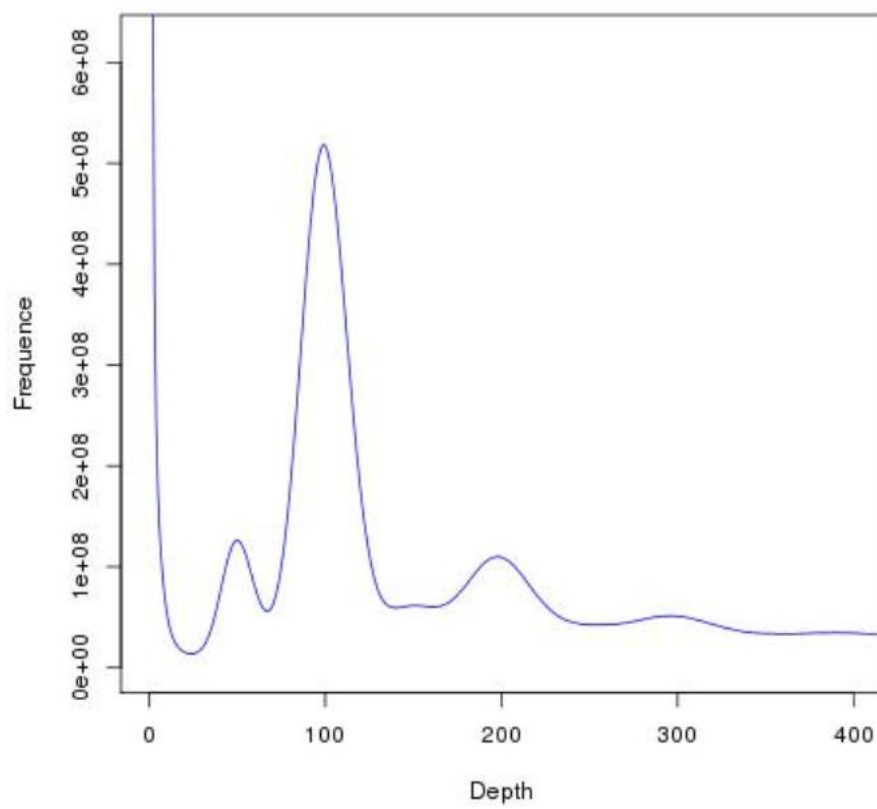

Supplemental Figure 1: Frequency distribution of 17-mer analysisfor the genome of *C. chinensis*.

Supplement: Supplementary file 2 — Supplemental Figure 1 [file 41438_2021_559_MOESM2_ESM.pdf]

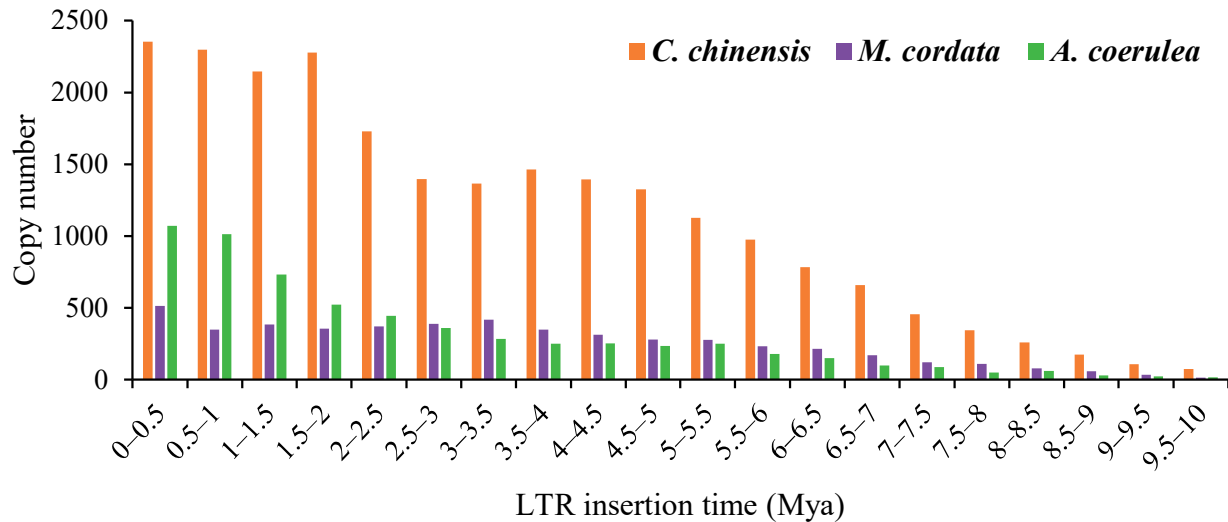

Supplemental Figure 4. Distribution of insertion times for LTRs.

Supplement: Supplementary file 5 — Supplemental Figure 4 [file 41438_2021_559_MOESM5_ESM.pdf]
