# Supplementary material for: The chromosome-level reference genome of Coptischinensis provides insights into genomic evolution and berberine biosynthesis
Source: Hortic Res. 2021 Jun 1;8:121. doi: 10.1038/s41438-021-00559-2 (PMC8166882; doi:10.1038/s41438-021-00559-2)
Supplement: Supplementary file 3 — Supplemental Figure 2 [file 41438_2021_559_MOESM3_ESM.pdf]

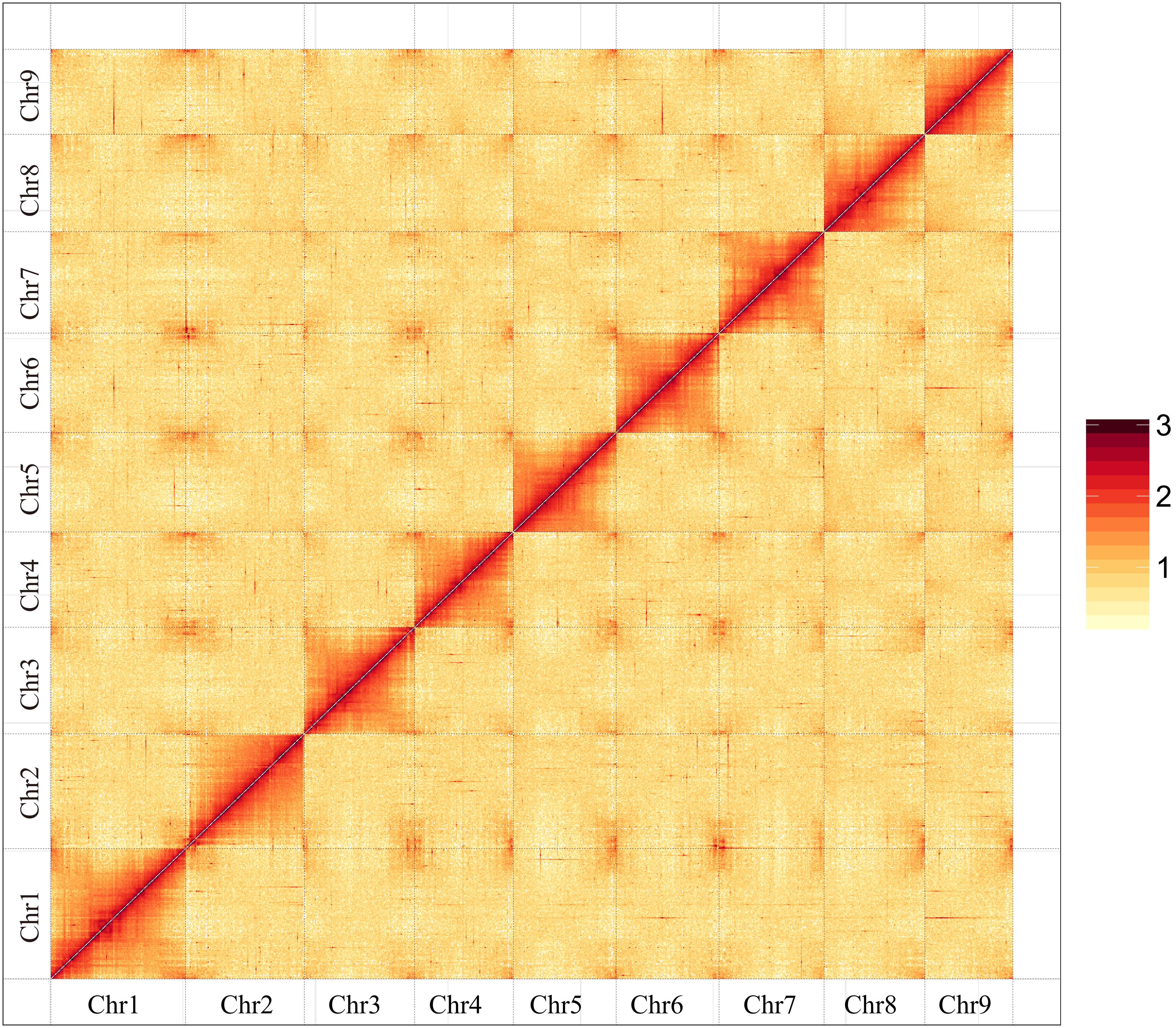

Supplemental Figure 2. Heat map of Hi-C interaction between the 9 chromosomes of *Coptis chinensis* genome.
